# The pedagogical relationship in medical education and training: A critical analysis

**DOI:** 10.3205/zma001704

**Published:** 2024-09-16

**Authors:** Eva Matthes, Thomas Rotthoff

**Affiliations:** 1 University of Augsburg, Faculty of Philosophy and Social Sciences, Pedagogy, Augsburg, Germany; 2 University of Augsburg, Faculty of Medicine, Medical Didactics and Education Research, DEMEDA, Augsburg, Germany

## Introduction

Having excellent medical expertise does not necessarily mean having the skills to effectively impart this knowledge to students. As a result, the didactic training of doctors is now a core component of faculty development programs. Good teaching quality arises not only from the proficient transmission of information but also fundamentally from the promotion of learning itself [1]. Reflecting on the importance of the pedagogical relationship in medical education and training can help in understanding how learning can be fostered. Currently, only a few empirical studies have explored the background and significance of pedagogical relationships in medical education and training [2], [3]. Against this backdrop and with the goal of further developing medical didactic training programs, we aim to stimulate a discussion on the pedagogical relationship through this contribution and an interdisciplinary exchange between medical didactics and pedagogy.

- What are the central features of pedagogical relationships?
- What is the role of the teacher?
- How should learners be perceived in this relationship?
- What role do the subjects being taught play?
- Does digitalization make teachers redundant?

Approaching these questions scientifically and seriously means critically engaging with widely held myths.

## Myth 1: The pedagogical relationship is an I-you relationship

This concept suggests that the relationship is only about the interactions between two individuals or two groups of individuals characterized by their personal dignity and mutual respect. However, the teaching-learning relationship is established to facilitate the acquisition of knowledge, skills, and attitudes; this implies that the teacher’s actions are not directly aimed at the learners (i.e., the “you”) but rather at their respective acquisition activities. Based on their existing knowledge, skills, and attitudes, teachers’ professionalism involves

1. critically evaluating the prescribed content to select what should be taught (knowledge, skills, etc.) and
2. to take care of target group-appropriate, and thus always differentiating, teaching methods in order to initiate, motivate, consolidate and continue appropriation processes through practice.

Ultimately, this approach helps individuals transition from externally guided learning processes to self-directed processes.

## Myth 2: The pedagogical relationship is a partnership

If a partnership is understood as being a symmetrical relationship – as the term itself suggests – then a pedagogical relationship is *not* a partnership but rather a relationship sui generis, which means unique in its characteristics [4]. Teachers and learners may face each other as equals in personhood, yet their relationships inherently involve an imbalance concerning what is to be learned and acquired. Put differently, a teaching-learning relationship is only established and makes sense if the teachers possess more knowledge and/or greater skills and/or clearer orientations/attitudes – ideally in combination – than the learners. Regarding the “third factor” [5], that is, what is to be learned and acquired, there is always asymmetry in teaching-learning relationships. However, this asymmetry aims to balance out during the teaching-learning process, not at cementing hierarchies. Because the processing of what is taught is always subjective and new insights can emerge during the learning it can never be ruled out that the teacher-learner relationship may be reversed after some time: Former learners can become the teachers of their former teachers. This is not a failure for the latter but, on the contrary, a great success. If someone can eventually teach something better than the teacher, it is not a defeat but a stroke of luck for the teacher and significant teaching success [6]. It also reminds us that teaching, if it is not to lead to an abuse of power, is always designed to end.

## Myth 3: Teaching is a natural talent or: You only have to have a will to teach

In public perception, the image of the “teacher as a natural talent” is still widespread. In addition, it is true that educators, even in medicine, should possess certain fundamental traits: empathy, openness, interest in people and things, and confidence. However, teaching is also a skill that can be taught through medical didactic offerings and faculty development programs. Therefore, we do not need to rely on natural talent. Currently, didactic training is often dominated by the teaching of skills for instruction and assessment. Competence models further examine several key dimensions that professional educators must possess: subject knowledge, subject-specific didactic knowledge, developmental psychological knowledge, pedagogical and general didactic knowledge, methodological and media knowledge, organizational pedagogical knowledge, and societal and political knowledge [7], [8]. Teaching is a highly demanding activity that needs to be learned and requires continuous professional development. It is not enough to be a “teacher at heart” [9]. The complexity of professional teaching is still underestimated, even in medicine. Teaching is not just about willingness; it requires ability, extensive knowledge, practice, reflection, and ongoing education.

## Myth 4: Subject competence equals teaching competence

A widespread belief is that someone who knows their subject well and has thorough expertise in it can also teach it effectively, implying that teaching competence is inherently tied to subject expertise. For a very long time, this notion has shaped and dominated the training of high school teachers and, to some extent, university education in Germany [10]. This perspective overlooks the necessary role shift from a subject specialist to an educator when the current task involves initiating and facilitating learning processes. Someone who wants to be a good, convincing, and effective teacher must be ready, willing, and able to undergo this transformation. In addition to successfully building pedagogical relationships, this requires, first, a willingness to engage with the selection criteria developed by general didactics, such as exemplarity, typicality, and representativeness, to present their content in an elemental (not trivialized) manner, thus making it comprehensible [11]. Second, methodological knowledge about the various means through which something can be taught is required so that different individuals can process and internalize the material while finding their own points of connection to absorb and productively engage with the content [12].

## Myth 5: Teachers are replaceable

In the 1920s, the educational theorist Herman Nohl called for a “passionate relationship” between teachers and students to help students “come into their own form” [4]. This concept has since faced significant criticism in educational discourse: It has been seen as leading to the unprofessional overburdening of teachers and an assumed omnipotence [13], [14]. Additionally, some interpretations of the term “passionate” suggest an erotic or even sexually abusive connotation [15]. In the context of digitization, there has been a – quite critically – observed idea that digital tools could be more important than teachers [16], [17]. Then, John Hattie came along, who, likely without reference to Nohl’s concept of a “pedagogical relationship”, emphasized not only engagement but also the necessity of passion in teaching with a contagious effect ([18], p.23). Passionate teaching, according to Hattie, requires more than just content knowledge and technical skills. It demands a love for the subject, an ethic of care, and a desire to instill a liking or even love for the subject in students ([18], p.24). Hattie, much like Nohl, clearly recognizes the triangular structure of the pedagogical relationship. For Hattie, the quality of a teacher’s actions is crucial. His concept has nothing to do with the idea of a born teacher. However, for him, all the threads of successful teaching come together with the teacher, making them irreplaceable because of their openness, flexibility, creativity, and critical thinking skills. Nonetheless, teachers must continuously seek professional development ([18], p.108-128), [19].

## Myth 6: Instructional teaching paralyzes student activity

In some parts of educational science, especially those aligned with constructivism, there are significant reservations about teaching and all forms of instruction. In this context, teaching is discredited as an intrusive act that stifles student activity or fails to sufficiently consider it. In contrast, Hattie concludes from his meta-study that “direct instruction” has an undeserved bad reputation. All the research findings show how effective this approach is ([18], p.204ff), [20]. A key feature of direct instruction is the guidance of the teaching process by the teacher. This type of teaching should not be confused with exclusively question-led frontal teaching. Rather, it is very demanding and offers students a variety of learning opportunities that the teacher oversees to ensure their proper use and benefit. In this sense, the teacher takes responsibility for both the fact that learning occurs and how it occurs.

According to Hattie, direct instruction consists of seven steps ([18], p.205f), [21]:

1. Clear objectives and success criteria that are transparent to the students.
2. Active involvement of the students in the learning tasks.
3. A precise understanding by the teacher of the methods and media to convey and explain the learning content.
4. Continuous assessment during the teaching process to ensure that students have correctly understood the material before moving forward.
5. Guided practice under the supervision of the teacher with individual feedback.
6. Summarizing the learned material in a way that is understandable to the students, integrating key thoughts or terms into a larger context.
7. Repeated practical application of the learned material in various contexts.

Only in this way can intensive learning that does not exhaust itself in superficial retention processes occur.

## Conclusion

A critical examination of the widespread myths about educational relationships clarifies that teachers not only convey content but also possess the ability to promote and support students’ learning processes within the framework of the educational relationship.

Some contributions to this issue of GMS Journal of Medical Education also emphasize the importance of the educational relationship in medical education. Schmidt et al. highlighted the importance of a trusting relationship between students and teachers through the development of a pilot curriculum for longitudinal professionalism development for medical students at the University of Jena [22]. In a qualitative study, Gehrke-Beck et al. described with a qualitative study the role of doctors in their further training as teachers working in medical practices [23]. The educational relationship is particularly important here because students find this relationship advantageous because of a perceived lower hierarchy and role model function. The study also underscores the need for better didactic preparation by teachers.

## Author’s ORCID

Thomas Rotthoff: [0000-0002-5171-5941]

## Competing interests

The authors declare that they have no competing interests. 
